# Canine Leishmaniasis in an Endemic Area for Human Leishmaniasis in Nicaragua

**DOI:** 10.1155/2022/5774296

**Published:** 2022-08-29

**Authors:** Byron Flores, Brenda Mora-Sánchez, Dayana Torres, Jessica Sheleby-Elías, William Jirón, José Luis Balcazar

**Affiliations:** ^1^Centro Veterinario de Diagnóstico e Investigación (CEVEDI), Departamento de Veterinaria y Zootecnia, Escuela de Ciencias Agrarias y Veterinarias, Universidad Nacional Autónoma de Nicaragua-León (UNAN-León), Carretera a La Ceiba 1 Km al Este, León, Nicaragua; ^2^Catalan Institute for Water Research (ICRA), Grahit, Girona 17003, Spain

## Abstract

In this study, the frequency of canines infected with *Leishmania* spp. in an area endemic to leishmaniasis in humans was determined. A descriptive pilot study was conducted between the months of October and December 2020 on dogs from Rota, a community in the municipality of León, which included 45 specimens from the peridomestic area. Different variables from each specimen were monitored, such as age, sex, breed, body condition, and clinical characteristics, as well as information on the owners and cases of human leishmaniasis presented in less than 5 years. Blood samples were collected from the cephalic vein and peripheral blood was separated. A complete blood count (CBC) was performed using venous blood samples with ethylene diamine tetraacetic acid (EDTA), as well as a conventional PCR was applied for the detection of *Leishmania* spp. Amastigotes were found in 22% of venous or peripheral blood samples, whereas a high prevalence of 28.89% (95% CI: 14.53–43.24) was found by PCR. Only 1/12 of positive dogs in PCR presented dry exfoliative dermatitis, therefore, there was no significant difference (*p* ≥ 0.05), the age and sex of the dogs were also not factors associated with infection (*p* ≥ 0.05). This study reports for the first time the molecular detection of *Leishmania* in dogs in an endemic area of leishmaniasis in humans in Nicaragua. The high frequency of dogs infected with Leishmania suggests that they play an important role in the transmission cycle of human leishmaniasis.

## 1. Introduction

Leishmaniasis represents a risk to public health, which is estimated to cause high morbidity and mortality in countries in tropical and subtropical zones [1]. However, this zoonotic parasitosis is still considered a neglected disease [2]. In humans, three classic forms are described: visceral (VL), cutaneous (CL), and mucocutaneous (MCL). Visceral leishmaniasis is endemic in Latin America, involving countries such as Bolivia, Colombia, Costa Rica, El Salvador, Guatemala, Honduras, Mexico, Nicaragua, Venezuela, and Brazil, with the latter showing the majority of cases [3]. Small flies of the species *Lutzomyia longipalpis* are the main vectors in the Americas [4, 5]. Canines are affected and are considered to be the main rural, peridomestic and domestic reservoir of *L. infantum* (syn. *L. chagasi*) associated mainly with VL in humans [6], although canine infections by *L. tropica*, *L. major*, and *L. braziliensis* have also been observed [6–9]. In fact, in some countries, millions of dogs have been euthanized as part of government policies to control human VL caused by *L. infantum*; however, some studies have also been published that question the effectiveness of this measure [10].

In Nicaragua, the species *L. braziliensi*, *L. panamensiscases* and *L. infantum* have been described in humans [6]. More than 90% of the cases of leishmaniasis in Nicaragua correspond to LC and LCM, with higher prevalence in the north and Atlantic area of the country, where values of up to 23.92% can be found (Municipality of El Cuá) [11]. Visceral Leishmaniasis is associated with *L. infantum* but it has been controlled in the country since no cases have been reported since 2011, however atypical cutaneous leishmaniasis is also caused by *L. infantum* [12], frequency has been detected in the Pacific Plain mainly in volcanic areas, including the communities of the municipality of León [11]. This atypical form is also known as the nodular or tuberculoid form of the disease and is characterized by papules and nodules in uncovered areas of the body and children are more often affected than adults. The cases of atypical cutaneous leishmaniasis in the country were 56 in 2016, 44 in 2018, and 19 in 2021 [13–15]. These atypical lesions are distinct from postkala-azar dermatitis, classical localized, or diffuse CL [2]. Although several studies related to the incidence and management of patients have been carried out, these have only been directed at human cases [2]. However, no studies of leishmaniasis in canines have been carried out; therefore, the current epidemiological situation in the country is not known. The aim of this study was to determine the frequency of canines infected with *Leishmania* spp. in an area endemic to atypical cutaneous leishmaniasis in humans, in order to provide epidemiological information and contribute to public health in Nicaragua.

## 2. Materials and Methods

A descriptive pilot study was conducted between the months of October and December 2020, on dogs from Rota, a community located in the volcanic reserve of the same name in the municipality of León. This community is considered endemic for atypical cutaneous leishmaniasis, which is located in the geographic coordinates: 12°32″0′ North and 86°43″0′ East and has a Tropical Savannah climate, with a height above sea level of 92.28 m, an annual rainfall of 1827 mm, and temperatures between 27–34°C.

For the study, a total of 45 canines were calculated from an unknown population, with a confidence level of 90%, an accepted error of 10%, and an expected prevalence of 17.3% [16]. These canines inhabited the peridomestic area of the study area.

Blood samples were collected from the cephalic vein with a 21G needle and a 5 mL syringe to extract 4 mL of blood, the sample was placed in a test tube with ethylene diamine tetraacetic acid (EDTA) to prevent coagulation. To take a peripheral blood sample, the area was shaved to perform a puncture with a sterile needle in the auricular vein, with a capillary the drop of blood was taken, which was then deposited on a slide to make the smear and was left to dry in the air.

Different variables from each animal were monitored at the time of taking the samples, applying a data collection form designed exclusively for this study. The variables included information related to demographic of the dogs (age, sex, race, body condition) as well as clinical characteristics. In addition, information was collected on the owners and the cases of human leishmaniasis that had occurred in less than 5 years.

### 2.1. Sample Analysis

From the venous blood samples with EDTA, a Complete Blood Count (CBC) was performed to associate the blood parameters with the clinic and the presence of *Leishmania* spp. [17].

The smears obtained from venous blood and peripheral blood were stained with Giemsa following the previously described protocol [18], and observation was carried out under the microscope with a 100 X objective lens.

For the molecular detection of *Leishmania* spp, 200 *μ*l of blood with EDTA was taken for DNA extraction with the QIAamp DNA Mini Kit, according to the manufacturer's instructions (QIAGEN; Germany). Conventional PCR was applied with the primers Hfor/Hrev (5′- CCTATTTTACACCAACCCCCAGT-3´/5′- GGGTAGGGGCGTTCTGCGAAA -3′) [19], in which a 120 bp fragment of the *Leishmania* kDNA is amplified, with more than 10,000 copies present in each parasite. The final reaction volume was 50 *μ*l, adding 25 *μ*l of Master Mix 2X (Promega, USA), 12 *μ*l of nuclease-free water, 4 *μ*l of each primer (1 × 10^3^ nM) and 5 *μ*l of DNA sample. The PCR reaction was performed with the Applied Biosystem 2720 Thermocycler, raising the temperature to 94°C for 10 minutes, followed by 40 cycles (95°C for 50 seconds, 55°C for 1 minute, 72°C for 1 minute), a final extension for 7 min at 72°C. As negative (water) and positive (*L. mexicana* culture) controls were also included. To visualize the PCR products, an electrophoresis was performed on a 1.3% agarose gel stained with ethidium bromide, applying 10 *μ*l of the product of amplification in each well. Visualization was performed in a UV light transilluminator.

### 2.2. Statistical Analysis

The results of the smear from peripheral and venous blood and the PCR are presented as a percentage with their respective 95% confidence intervals (CI:95%), while in the comparison of the results between the tests, the test was applied McNemar and Kappa concordance was determined. In the bivariate analysis for the identification of factors, the Chi square test was applied.

## 3. Results

**Figure 1:**
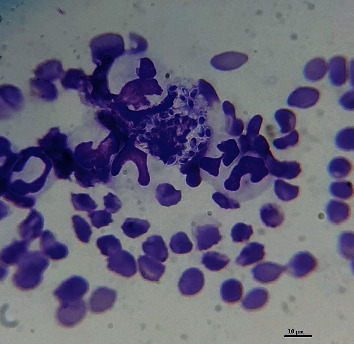
Amastigote of *Leishmania* ssp. in blood smears stained with Giemsa, 100 X.

**Figure 2:**
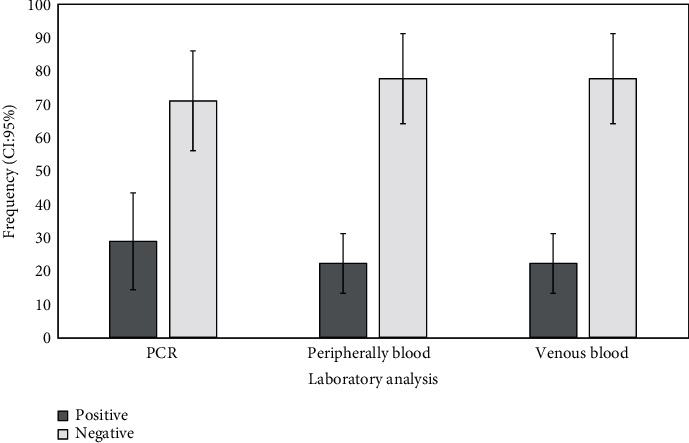
Percentage frequency of positive canines in PCR and smears from venous and peripheral blood.

The PCR analyses revealed that 13/45 sampled canines were found be positive, which represented a prevalence of 28.89% (95% CI: 14.53–43.24). In the samples analyzed in venous blood smears, as well as in those of peripheral blood, the parasite was observed in 10/45 samples, which represented a prevalence of 28.89% 22.22% (9% CI: 8.96–35.48) for both cases (Figure 1). A comparison of the frequency of positive samples using the three diagnostic techniques (PCR, venous blood smear, peripheral blood smear) revealed no significant differences (*p*=0.5811) (Figure 2).

The positive frequency in at least one of the three techniques used showed that 13/45 of the dogs were positive, which represented a prevalence of 28.89% (95% CI: 14.53–43.24). The concordance analysis between the results of PCR and peripheral blood smear showed that 5 samples were positive in both tests, and 8 samples were positive in PCR, but negative in the peripheral blood smear. In addition, 5 PCR-negative samples were also negative in the smear, which gave a Kappa value of 0.245 (*p*=0.095). On the other hand, the comparison between PCR and venous blood smear revealed that 10 samples were positive for both tests; however, 3 PCR-positive samples were negative in the venous blood smear, which gave a Kappa value of 0.826 (*p* < 0.01) (Table 1).

The clinical characteristics of dogs showed that only 1/12 were positive in PCR when it came to dry exfoliative dermatitis, therefore, there was no significant difference (*p* ≥ 0.05), the age and sex of the dogs were also not factors associated with infection (*p* ≥ 0.05). Comparison of hematological parameters between canines infected and uninfected with *Leishmania* spp. revealed that the platelet count was 29076.923 cells/mm^3^ in uninfected dogs, a value significantly higher than the average of 18156.250 cells/mm^3^ found in dogs with the parasite (*p* < 0.05).

No significant differences (*p* ≥ 0.05) were found when comparing leukocyte count, erythrocyte count, hematocrit, percentage of neutrophils, band neutrophils, lymphocytes, monocytes, eosinophils, and basophils (Table 2).

**Figure 3:**
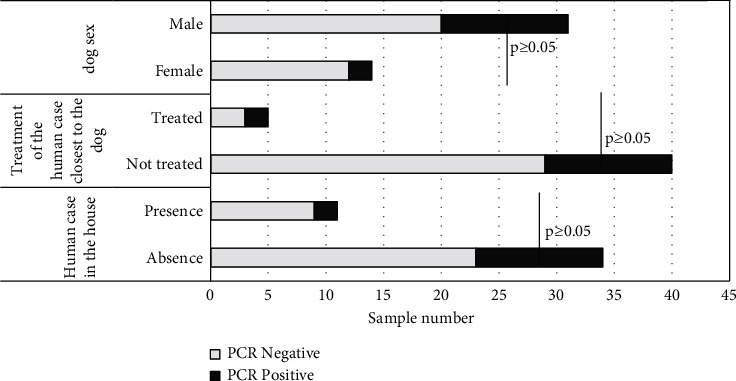
Bivariate comparison between risk factors and infection by *Leishmania* spp.

A comparison between the cases in dogs and humans revealed that of the 2/13 dogs infected with *Leishmania* spp, they lived in a house where leishmaniasis had been diagnosed in humans. The bivariate analysis for the identification of factors associated with *Leishmania* spp. infection in canines revealed that the sex of the animal, living in the same house with a human case, as well as the antiparasitic treatment of the human case closest to the dog, were not associated with the result of PCR (*p* ≥ 0.05) (Figure 3).

## 4. Discussion

Leishmaniasis is a spectrum of neglected vector-borne diseases caused by different species of protozoan parasites of the genus *Leishmania*. It is considered that in urban areas the dog is the main reservoir of the parasite and a key element in the epidemiological cycle. Therefore, the detection of canine infection is crucial to providing reliable data to promote and support One Health programs [20]. In this study, canines from a community endemic to human atypical cutaneous leishmaniasis were examined using PCR, which showed a high prevalence of infected dogs (28.89%) as compared to other studies carried out in other endemic areas, such as Venezuela, where only 5.3% of positive dogs were detected [21]; however, it was lower than that observed in the north of Brazil, one of the countries most affected by this parasitosis, in which they found a prevalence in dogs (54.7%) applying conventional PCR in blood [22]. This study demonstrates that the transmission of leishmaniasis to humans is high in this area, which reinforces the important role that dogs play in the endemic cycle of leishmaniasis, whose main strategies include timely diagnosis and treatment of human cases, environmental management, chemical control of the vector with residual insecticide sprays, and canine serological surveillance [23]. Tools to prevent *L. infantum* infection in dogs include the use of topical insecticides, insecticide-impregnated collars, and vaccinations [24]. Recent now allowing veterinarians to treat infected seropositive dogs with miltefosine as an alternative to euthanasia [10], it has been observed that the use of miltefosine administered orally for 4 weeks contributes to a clinical improvement and reduction in infectivity of dogs to *L. infantum* [4]. Furthermore, vaccination has also been proposed for parasite control in dogs, though there is no strong scientific evidence to support the idea that it can reduce transmission from infected dogs to sandflies, which would significantly reduce the risk of infection by *L. infantum* in humans [25].

In smear stain venous blood samples, amastigote was found in 22%, this is a high frequency, because the technique has low sensitivity and because in veterinary medicine parasites are rarely observed in blood smears [26], As described, amastigotes were found in only 0.3% of dogs with leishmaniasis, both free and within circulating leukocytes (neutrophils, monocytes, macrophages) [27]. In this study, blood samples were taken, however, other studies have shown that the diagnosis is more sensitive when samples of bone marrow or lymph nodes are analyzed [26, 28]; therefore, the prevalence could be higher than that observed, however, taking these types of samples, they are very traumatic.

Compared to the clinical symptomatology of the dogs, there was no significant difference in the PCR results (*p* ≥ 0.05), indicating that canines in an endemic area present a much higher prevalence of infection than the proportion that actually develops the disease and can also be a source of infection for the sandfly [29, 30].

In the case of skin lesions, desquamative dermatosis was observed in 4.4% of the dogs and weakness and alopecia in 25%, a low frequency when compared to the study carried out by [31] in which they found in the seropositive population, 75% of dogs had skin ulcers, alopecia, and onychogryphosis.

When comparing the hematological parameters between the positive and negative dogs in the PCR, we only found significant differences in the platelet count; however, a different finding was described by [32], who observed a significant decrease in the number of red blood cells, hemoglobin and packed cell volume in Leishamania-infected dogs. This could be attributed to the fact that infected dogs have red blood cells with a shorter half-life associated with a change in the fluidity of membrane lipids after oxidative stress [33]. This study found that only 7.7% of dogs infected with *Leishmania* were symptomatic, so the lack of association between hematological values and infection may be explained by the fact that asymptomatic dogs exhibit erythrocyte counts, hemoglobin values, and hematocrits higher than symptomatic dogs which may be related to a high bone marrow parasite load, which is associated with severe leishmaniosis [34]. The lower platelet count in infected dogs is explained by [35] in a mouse model of experimental visceral leishmaniasis, who observed a progressive decrease in platelets from day 14 postinfection, culminating in severe thrombocytopenia on day 28. Plasma thrombopoietin levels were reduced in infected mice, at least in part because of alterations in the hepatic microenvironment associated with granulomatous inflammation.

## 5. Conclusions

This study reports for the first time the molecular detection of *Leishmania* in dogs in an endemic area of leishmaniasis in humans in Nicaragua. The high frequency of dogs infected with Leishmania suggests that they play an important role in the transmission cycle of human leishmaniasis. Therefore, the dynamics of the canine population should be considered in the epidemiological surveillance of leishmaniasis in low- and middle-income countries.

## Figures and Tables

**Table 1:** Comparison of tests for the detection of dogs infected with *Leishmania* spp.

		Peripheral blood smear		Venous blood smear	
		Negative	Positive	Negative	Positive
PCR	Negative	27	5	32	0
	Positive	8	5	3	10
	Total	35	10	35	10
	Kappa	0.245		0.826	
	Significance	0.095		0.000	

**Table 2:** Comparison of hematological parameters in canines according to the diagnosis of *Leishmania* spp.

Hematological parameter	PCR	Mean	Standard deviation	Significance	Mean difference	95% CI for the difference in means	
						Lower	Upper
Leukocyte count (cel./mm^3^)	Negative	3846.88	1924.75	0.804	−160.82	−1458.98	1137.35
	Positive	4007.69	2038.57				

Erythrocyte count (cel./mm^3^)	Negative	1104062.50	815403.56	0.963	11754.81	−497594.42	521104.04
	Positive	1092307.69	628876.69				

Platelet count (cell/mm^3^)	Negative	**29076.92**	**15971.13**	**0.044**	**10920.67**	**21509.63**	**331.71**
	Positive	**18156.25**	**15961.87**				

Hematocrito (%)	Negative	34.19	11.15	0.159	−4.97	−11.96	2.02
	Positive	39.15	8.77				

Neutrophil (%)	Negative	27.80	19.36	0.266	−7.53	−21.01	5.94
	Positive	35.33	22.61				

Band neutrophils (%)	Negative	10.75	9.72	0.859	0.56	−5.79	6.92
	Positive	10.19	9.22				

Lymphocyte (%)	Negative	38.13	18.23	0.099	9.55	−1.87	20.97
	Positive	28.58	14.30				

Monocyte (%)	Negative	15.81	12.41	0.482	2.97	−5.47	11.41
	Positive	12.84	13.51				

Eosinophil (%)	Negative	2.91	3.62	0.11	−2.28	−5.08	0.53
	Positive	5.19	5.51				

Basophil (%)	Negative	4.35	4.79	0.464	−1.25	−4.67	2.16
	Positive	5.60	5.98				

Bold values are significant (*p* < 0.05).

## Data Availability

All data generated or analyzed during this study were included in this published article; thus, no additional data were available.
